# Clozapine: An Updated Overview of Pharmacogenetic Biomarkers, Risks, and Safety—Particularities in the Context of COVID-19

**DOI:** 10.3390/brainsci10110840

**Published:** 2020-11-11

**Authors:** Ana Miruna Dragoi, Ioana Radulescu, Bogdana Adriana Năsui, Anca Lucia Pop, Valentin Nicolae Varlas, Simona Trifu

**Affiliations:** 1 Department of Psychiatry, “Alexandru Obregia” Clinical Hospital for Psychiatry, 10 Berceni St., 041914 Bucharest, Romania; anamirunadragoi@gmail.com; 2 Department of General Medicine, “Carol Davila” University of Medicine and Pharmacy, 37 Dionisie Lupu St., 020021 Bucharest, Romania; ioana.radulescu95@gmail.com (I.R.); varlas.valentin@umfcd.ro (V.N.V.); 3 Department of Community Health, “Iuliu Hațieganu” University of Medicine and Pharmacy, 6 Louis Pasteur St., 400349 Cluj-Napoca, Romania; nasuibogdana@yahoo.ro or; 4 Department of Clinical Laboratory, Food Safety, “Carol Davila” University of Medicine and Pharmacy, 6 Traian Vuia St., 020945 Bucharest, Romania; 5 Department of Clinical Neurosciences, “Carol Davila” University of Medicine and Pharmacy, 37 Dionisie Lupu St., 020021 Bucharest, Romania; simona.trifu@umfcd.ro

**Keywords:** clozapine, schizophrenia, pharmacogenetic, early onset, pregnancy, bipolar affective disorder, agranulocytosis, Romania, COVID-19

## Abstract

Background: clozapine (CLZ) use is precarious due to its neurological, cardiovascular, and hematological side effects; however, it is the gold standard in therapy-resistant schizophrenia (TRS) in adults and is underused. Objective: to examine the most recent CLZ data on (a) side effects concerning (b) recent pharmacological mechanisms, (c) therapy benefits, and (d) the particularities of the COVID-19 pandemic. Data sources: a search was performed in two databases (PubMed and Web of Science) using the specific keywords “clozapine” and “schizophrenia”, “side effects”, “agranulocytosis”, “TRS”, or “bipolar affective disorder (BAF)” for the last ten years. Study eligibility criteria: clinical trials on adults with acute symptoms of schizophrenia or related disorders. Results: we selected 37 studies, randomized controlled trials (RCTs), and clinical case series (CCS), centered on six main topics in the search area: (a) CLZ in schizophrenia, (b) CLZ in bipolar disorder, (c) side effects during the clozapine therapy, (d) CLZ in pregnancy, (e) CLZ in early-onset schizophrenia, and (f) CLZ therapy and COVID-19 infection. Limitations: we considered RCTs and CCS from two databases, limited to the search topics. Conclusions and implications of key findings: (a) clozapine doses should be personalized for each patient based on pharmacogenetics testing when available; the genetic vulnerability postulates predictors of adverse reactions’ severity; patients with a lower genetic risk could have less frequent hematological monitoring; (b) a CLZ-associated risk of pulmonary embolism imposes prophylactic measures for venous thromboembolism; (c) convulsive episodes are not an indication for stopping treatment; the plasma concentration of clozapine is a better side effect predictor than the dosage; (d) COVID-19 infection may enhance clozapine toxicity, generating an increased risk of pneumonia. Therapy must be continued with the proper monitoring of the white blood count, and the clozapine dose decreased by half until three days after the fever breaks; psychiatrists and healthcare providers must act together.

## 1. Introduction

Controversies are frequent in psychiatric therapy, and consensus is hard to find [1]; however, there is widespread agreement regarding the exclusive role of clozapine in treating severe refractory schizophrenia [2]. Schizophrenia is a major mental illness with a lifelong impact on patients and their caregivers. The precise etiopathology of schizophrenia is unspecified and most probably multifactorial [3], implying neurodevelopmental (hypoxia, maternal infection, and stress), genetic (family history), and environmental factors (social and cannabis use) [4].

Second-generation antipsychotics (SGAP) are the first line of treatment for acute psychotic episodes and are currently prescribed for long-term management of schizophrenia, affective disorders, and some dementia-related symptoms; SGAP are considered atypical when comparing their clinical profile with first-generation antipsychotics and respond better to the negative symptoms of schizophrenia. Extrapyramidal side effects are less common than with typical ones [5,6]. Risperidone, ziprasidone, paliperidone, and aripiprazole are potent D2 dopamine receptor antagonists; quetiapine and clozapine are weak D2 antagonists and antagonists for 5-HT 2A, as well as agonists for 5-HT 1A receptors.

The most potent molecules that bind to the alpha-adrenergic receptors are clozapine, iloperidone, and risperidone. Clozapine, olanzapine, and quetiapine also bind to muscarinic cholinergic receptors [7,8]. The less common incidence of extrapyramidal side effects has made atypical antipsychotics very popular among psychiatrists [9]. However, they still carry a risk of side effects that must be monitored, including metabolic disorders (type 2 diabetes, weight gain, dyslipidemia) and cardiovascular disorders like the prolongation of the QT interval on Electrocardiogram [10], as well as neurological and hematological (agranulocytosis) complications.

The life expectancy of patients diagnosed with schizophrenia and affective bipolar disorder is between 11 and 20 years shorter, as patients are vulnerable and in continuous need of medical and social care to prolong their life expectancy [11]. One-third of patients respond to “typical” antipsychotics (e.g., chlorpromazine and haloperidol) [12]; the remaining two-thirds need a second strategy. Clozapine is established as the gold-standard treatment for treatment-resistant schizophrenia (TRS): 32% of short-term and almost 40% of long-term therapy TRS patients respond to clozapine [13,14,15]; the absolute reduction in overall positive and negative symptom scale (PANSS) scores is clinically significant.

Considering the terrible burden that schizophrenia places on patients and their families, the discovery of clozapine, the first atypical antipsychotic, was a substantial pharmacological and clinical milestone. The significant therapeutic effect of clozapine compared with other classes of drugs and the reduced incidence of extrapyramidal side effects, which increased the stigma on psychiatric patients, and brought hope in the most severe cases of schizophrenia.

At the moment, clozapine is the most effective antipsychotic drug for therapy-resistant schizophrenia (TRS) [16,17,18], listed on the WHO Model List of Essential Medicines [19] and superior to other drugs in the class due to: (1) a lower risk of suicide, (2) lower risk for tardive dyskinesia, (3) the improvement of cognition and improved quality of life, and (4) the decreased risk of relapse. Clozapine is specific to psychiatric therapy due to its effectiveness but also due to a pharmacodynamic conundrum. Even though it had a rising prescription trend in 2005–2014 with a relative increase of 7.8%, up to 197.2%, clozapine (CLZ) remains underused due to its specific adverse reactions: hematological (agranulocytosis), cardiovascular, and neurological side effects. Despite the benefits, clozapine remains underutilized in up to two-thirds of TRS cases in most countries, as revealed in an Australian study in 2017 [20]. Less is known about clozapine use in Romania.

An update for the actual clinical experience will reinforce the therapy benefit, considering the increased safety of use due to screening, the early detection of side effects, and the decision to treat patients within the margins of safety and not underuse the drug.

The purpose of this review was to examine the latest research regarding: (a) potential pharmacogenetic marker predictors of adverse reactions associated with clozapine treatment, registered in the last ten years; (b) side effects not as isolated events, but as a network of interdependent elements managed as a whole; (c) use in younger patients or during pregnancy; (d) strategies in clozapine resistance pathology; and (e) particularities of clozapine therapy in the COVID-19 pandemic.

## 2. Materials and Methods/Data Search

In the present paper, we performed a systematic qualitative review according to the Preferred Reporting Items for Systematic Review and Meta-Analysis (PRISMA) guidelines searching original published papers on clozapine use in humans, with a data filter on the current use of clozapine in younger patients, during pregnancy, about a bipolar spectrum disorder or COVID-19, and associated side effects; other criteria included: published in a scholarly peer-reviewed journal; written in English, French, or Romanian (but with no country restriction); and from the last ten years.

The review methods of the search were established beforehand. The initial review protocol assumed a Google Scholar search; due to document types’ diversity, the search was performed in two major databases, PubMed^®^/MEDLINE, and Web of Science Core Collection. The report had no other significant deviations from the initial study plan.

Information sources: we searched the databases PubMed^®^/MEDLINE (http://www.ncbi.nlm.nih.gov/pubmed) and Web of Science for clozapine, side effects, and related keywords. Search: we did a search in the two databases (filters applied: clinical trial, randomized controlled trial, clinical case series, on humans, in the last ten years) with the keywords: “clozapine” AND “side effects” OR “schizophrenia” OR “bipolar affective disorder” OR “TRS” OR “treatment-resistant bipolar disorder” OR “agranulocytosis” OR “obesity or metabolic” OR “pharmacogenetic” OR “pulmonary embolism” OR “seizures” OR “COVID-19” OR “pregnancy” OR “early-onset schizophrenia” OR “Romania”. We restricted searching for articles written in English, French, or Romanian; the last updated search was done on 15 October 2020.

Study selection: inclusion criteria were: (1) patients with a diagnosis of schizophrenia or a related disorder, on clozapine therapy or with an indication of (2) clozapine therapy; (3) non-pregnant or (4) pregnant adult or (5) child, in early-onset schizophrenia—compared to control or other antipsychotics; (6) patients on clozapine therapy with COVID-19 AND related side effects (neutropenia, agranulocytosis, pneumonia, pulmonary thromboembolism, seizures, obesity, and/or weight gain) due to therapy registered in randomized clinical trials (RCTs) and clinical case series (CCS) published in the last ten years.

Data extraction: the following data were selected: author(s), year of publication, country and aim of the study, study design, and main results. We selected RCTs and CCS as presented below for all the data searched and retrieved from the database sources. Two independent investigators extracted the data and selected a sample of eligible studies, achieving good agreement. Firstly, the authors screened articles by title and abstract, and then by full text. We did snowball searches of key papers. Duplicates and articles not fulfilling the search criteria were excluded.

Data analysis was performed by three authors (A.M.D., A.L.P., and B.A.N.). Over 3000 studies with schizophrenia spectrum disorder or bipolar spectrum disorders in therapy with clozapine, with or without a control group, were identified and screened for eligibility by the two examinators. According to the topic search, the data extracted included demographic variables, number of participants in the study, treatment, side effects profile, and associated comorbidities. We completed the data collection in October 2020. The quality of the studies selected for review was evaluated.

Thirty-seven papers were included in the present study, centered on the seven main topics included in the search. Statistical analysis was performed using Microsoft Excel^®^ 2013 (Microsoft^®^ Corporation, Redmond, WA, USA).

## 3. Results

### 3.1. Study Selection

#### 3.1.1. PubMed^®^/MEDLINE

**Figure 1:**
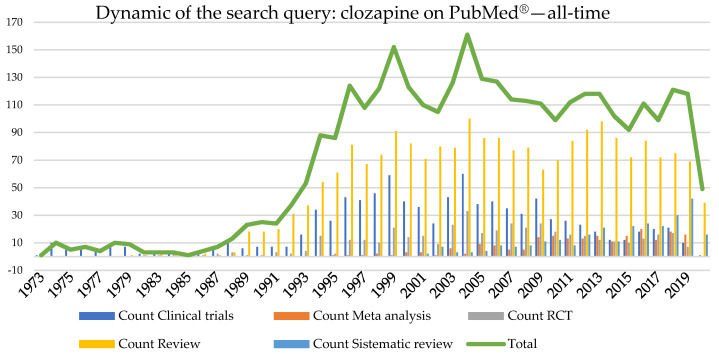
A systematic search for the keyword “clozapine” on the PubMed^®^ database (all-time topic) retrieved over 3075 results in total (RCT—Randomised Clinical Trials).

**Figure 2:**
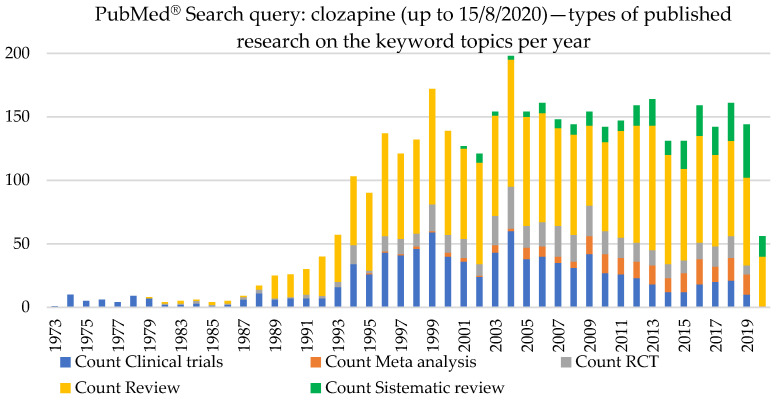
A systematic search for the keyword “clozapine” on the PubMed^®^ database (all-time topic) retrieved over 3075 results by type of study.

The “clozapine” keyword search (from inception until 15 August 2020) retrieved over 3075 results (Figure 1), 937 clinical trials, 211 meta-analyses, 277 systematic reviews, and 428 randomized clinical trials (Figure 2), showing a persistent interest in the widening of the use of clozapine as no alternative therapies are available yet.

For the association between “CLZ” and “schizophrenia”, the search refined to RCT/CT retrieved 127 results; “CLZ” and “treatment-resistant schizophrenia” retrieved 19 results, “CLZ” and “resistance” retrieved four results, “CLZ” and “bipolar disorders” retrieved 38 results, “CLZ” and “treatment-resistant bipolar disorder” retrieved seven results, “CLZ” and “agranulocytosis” retrieved 68 results, “CLZ” and “pharmacogenetic” retrieved six results, “CLZ” and “obesity” retrieved 12 results, “CLZ” and “pulmonary embolism” retrieved seven results, “CLZ” and “seizure” retrieved 23 results, “CLZ” and “COVID-19” retrieved five published papers, “CLZ” and “pregnancy” retrieved seven results, and “CLZ” and “early-onset schizophrenia” retrieved 12 results, and “CLZ” and “Romania” (TOPIC) retrieved three results.

#### 3.1.2. Web of Science

We searched for: TITLE: (Clozapine) Refined by TITLE: (side effects) AND DOCUMENT TYPES: (ARTICLE OR REVIEW) Timespan: 2010–2020. Indexes: Science Citation Index Expanded (SCI-EXPANDED), Emerging Sources Citation Index (ESCI), Current Chemical Reactions (CCR)-EXPANDED, Index Chemicus (IC). Results: 794 published papers (595 articles and 199 reviews).

For the association “CLZ” and “schizophrenia”, a search retrieved 21 results; “CLZ” and “treatment-resistant schizophrenia” and “CLZ” and “resistance” retrieved 21 results, “CLZ” and “bipolar disorders” retrieved 38 results, “CLZ” and “treatment-resistant bipolar disorder” retrieved seven results, “CLZ” and “agranulocytosis” retrieved 68 results, “CLZ” and “pharmacogenetic” retrieved six results, “CLZ” and “obesity” retrieved 12 results, “CLZ” and “pulmonary embolism” retrieved seven results, “CLZ” and “seizure” retrieved 23 results, “CLZ” and “COVID-19” retrieved five published papers, “CLZ” and “pregnancy” retrieved seven results, “CLZ” and “early-onset schizophrenia” retrieved 12 results; and “CLZ” and “Romania” (TOPIC) retrieved three results.

**Figure 3:**
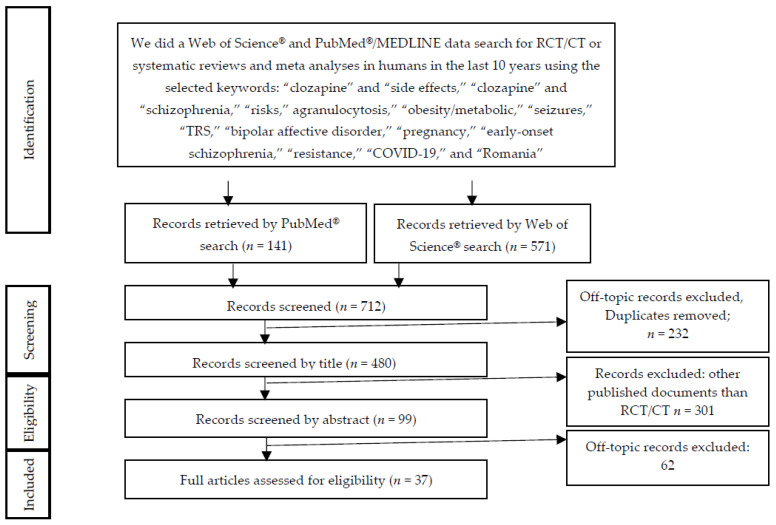
Preferred Reporting Items for Systematic Review and Meta-Analysis (PRISMA) diagram describing a systematic search and study selection process (RCT—Randomised Clinical Trials, CT—Clinical Trials).

From the databases, retrieving 99 articles initially screened by title and abstract or in extenso to match the search criteria, and excluding duplicates, we selected 37 studies for full-text reading, centered on six main topics in the research area: (a) treatment-resistant schizophrenia, (b) use in bipolar disorder, (c) side effects during clozapine therapy—agranulocytosis, metabolic side effects, pharmacogenetic severity markers, dysmetabolic side effects, pulmonary embolism, and seizure risk, (d) safety of clozapine in particular situations like pregnancy and early-onset schizophrenia, (e) clozapine resistance and electroconvulsive therapy (ECT) augmentation, and (f) clozapine therapy and COVID-19 infection. More detailed information regarding the selection process is presented in the PRISMA flow diagram (Figure 3, Table S1).

### 3.2. Clozapine

**Figure 4:**
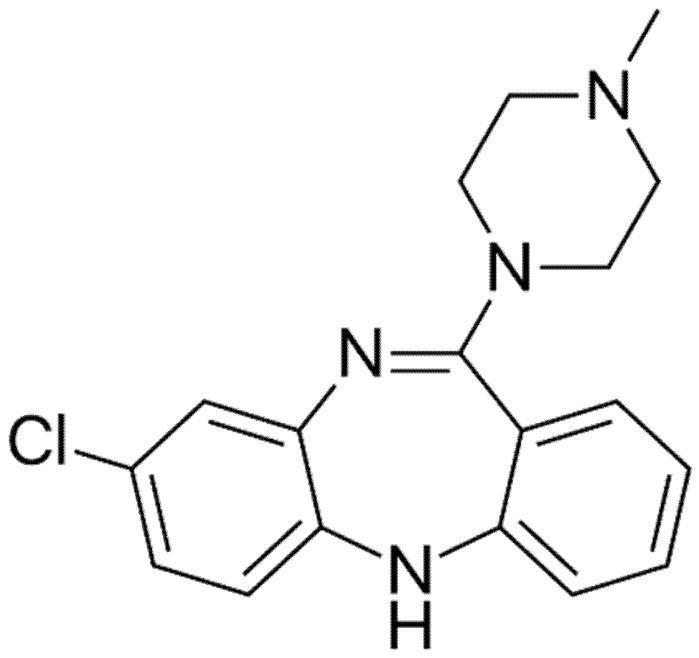
Clozapine chemical structure (IUPAC-C 3-chloro-6-(4-methylpiperazin-1-yl)-11H-benzo[b][1,4]benzodiazepine) [4].

Clozapine C_18_H_19_ClN_4_ (Figure 2) is the only atypical antipsychotic agent approved to manage treatment-resistant schizophrenia. From a pharmacological point of view, clozapine is a tricyclic dibenzodiazepine that binds to several receptors of the central nervous system (Figure 4).

This antipsychotic agent has a unique profile for binding to 5-HT 2A/2C receptors, being a serotonin antagonist. Clozapine also has an affinity for dopaminergic receptors, but it has only a weak antagonistic effect on the dopamine D2 receptor (known to modulate neuroleptic effects).

The altered schizophrenia mechanisms may imply non-dopaminergic pathways, mainly for TRS, involving the GABA-ergic system [21,22]. The direct interaction of clozapine with the GABA_B_ receptor was attested to by the X-ray structure and molecular docking of clozapine on the extracellular part of the GABA_B_ receptor [23].

The Wander Pharmaceutical Company synthesized clozapine in 1956 in Switzerland. In the mid-1960s, clozapine became part of pharmacodynamic research in Berlin and was recognized as unique due to its bidirectional effect as a neuroleptic and antipsychotic/antidepressant, as proven by the clinical studies [24].

More clinical studies showed clozapine’s antipsychotic properties and the safer profile regarding extrapyramidal side effects, so in the 1970s, it was released on the European market. It was soon withdrawn from pharmacies after some Finnish psychiatrists reported seven deaths related to a high incidence of agranulocytosis among elderly patients treated with clozapine [25,26]. In 1990, clozapine became available again in therapy, with a strict blood concentration monitoring protocol.

A short time later, two other second-generation antipsychotic agents were introduced: risperidone and olanzapine, compounds whose administration did not reveal any associated hematological risks, but none have shown efficacy for TRS.

The indications for the use of clozapine in therapy are treatment-resistant schizophrenia (TRS) and suicidal behavior in schizophrenia/schizoaffective disorder. A series of off-label uses are mentioned in the references: treatment-resistant bipolar disorder (TRBD) and psychosis/agitation associated with dementia, and psychosis in Parkinson’s disease [27].

However, at the moment, clozapine’s prescribing and monitoring regulations vary widely worldwide [28]. In most countries evaluated for regulatory reasons—China, Denmark, Ireland, Japan, the Netherlands, New Zealand, Romania, the UK, and the USA [28]—there is a mandatory neutrophil monitoring registrar, and the dispensing of clozapine is dependent on a minimal level of white cells and neutrophil count (except to Romania, Denmark, and the Netherlands). In the USA, the risk of agranulocytosis is strictly evaluated. The risk of severe neutropenia from clozapine is ~1.3% overall, with a peak at one month and a reduction in risk after more than one year [29]. Neutropenia generates a susceptibility to infections at absolute neutrophil counts (ANC) <500 μL; the COVID-19 pandemic promotes neutrophil counts on point-of-care devices [30]. Guidelines in New Zealand suggest echocardiography and routine troponin screening after the initiation of clozapine.

### 3.3. Pharmacogenetic Severity Markers as Potential Biomarkers in Clozapine Therapy

The current pharmacological doses are estimates for a standard patient; however, pharmacologists have highlighted two different genetic types: slow metabolizers and fast metabolizers. The type of metabolizer is generated by personal (genetic and metabolic) and environmental factors. Thus, when estimating the drug clearance (concentration-to-dose (C/D) ratio), a low C/D ratio indicates a fast metabolizer, while a high C/D ratio indicates a slow metabolizer. Clozapine C/D ratios range from 0.6 to 1.2 ng/mL per mg/day in the USA [31], with a double value in East Asians. Clozapine C/D ratios can be enhanced in interaction with inhibitors (including fluvoxamine and oral contraceptives) or an inflammatory state.

**Figure 5:**
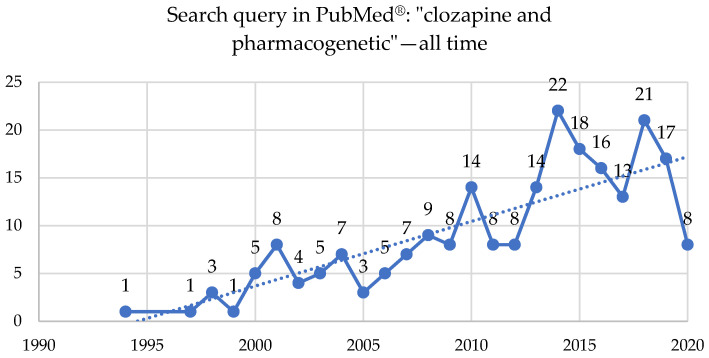
A search query in PubMed^®^: “clozapine” and “pharmacogenetic”—all time, up to 15 August 2020.

Two hundred and four studies were published on clozapine and pharmacogenetics topics in the PubMed^®^ database starting in 1994, with 57 reviews, three systematic reviews or meta-analyses, and 12 clinical trials or RCTs (Figure 5, Table S2).

The genetic vulnerability is correlated with metabolic side effects with a higher prevalence of adverse metabolic reactions in clozapine-treated patients and postulates predictors of severity—pharmacogenetics markers such as receptors CYP2C19, LEP, LEPR, and HTR2C [32]. Clozapine’s metabolism, elimination, and response were evaluated by genotyping specific enzymes, such as CYP1A2 and CYO2C19, and measuring HTR2C serotonin receptors leptin receptor, taking into account concomitant medication if present. The results showed that metabolic syndrome was correlated with higher levels of clozapine and CYP2C19*2 and leptin receptor G alleles. Individuals who metabolize CLZ more slowly are at higher risk for this type of disorder.

Antipsychotic-related weight gain is linked with several variants from nine genes (Adrenoceptor (ADR) Alpha-2A, Beta-3 (ADRB3), Brain-Derived Neurotrophic Factor (BDNF), Dopamine Receptor D2 (DRD2), Guanine Nucleotide-Binding Protein (GNB3), 5-HT (Serotonin) Receptor 2C (HTR2C), Insulin-induced gene 2 (INSIG2), Melanocortin-4 Receptor (MC4R), and Synaptosomal-associated protein, 25kDa (SNAP25) [33].

Gressier et al. (2015) found three genetic variants linked to clozapine response in serotonin genes, rs6313 and rs6314 within the HTR2A gene, rs1062613 within the HT3A gene, suggesting a possible serotonergic modulation of clozapine clinical response but no link with weight gain [34].

DiGeorge syndrome (22q11.2 deletion syndrome) with a 1/4000 live births prevalence [35], is associated with high frequencies of attention-deficit/hyperactivity disorder (ADHD), psychotic disorders, and eating, mood, and anxiety disorders. The rates of schizophrenia spectrum disorders in adults with 22q11.2DS over 25 are up to 41% [36,37]. Much of the published literature on 22q11.2DS is on treatment-resistant schizophrenia; therapy with clozapine is the gold standard, but the side effects of CLZ seem to be more frequent.

In clinical practice, pharmacogenetic testing is widely available, and psychiatrists should adjust and personalize clozapine doses for each patient, decreasing the risks of metabolic side effects to a minimum.

### 3.4. Treatment-Resistant Schizophrenia

A total of 1422 studies were published on clozapine and treatment-resistant schizophrenia on the PubMed^®^ database, of which 554 were from the past ten years, with four randomized controlled trials and ten systematic reviews and meta-analyses.

Treatment-resistant schizophrenia is described as the persistence of symptomatology after two trials of two different antipsychotic drugs of appropriate dosage and duration, with proven medication compliance [38]. Although persistent symptoms may be from any of the three areas of the disorder (negative, positive, cognitive), treatment-resistant schizophrenia is usually characterized by persistent positive symptoms [39].

The lack of response to antipsychotic medication is not enough to diagnose treatment-resistant schizophrenia, as clinicians must differentiate it from pseudo resistance.

The efficacy of clozapine was superior to chlorpromazine in resistant schizophrenia and to three previous antipsychotic drugs, as shown in a pivotal study (the U.S. Clozaril study) in a six-week trial on patients [40] that led to the FDA approval of clozapine for TRS but not as a first-line treatment due to side effects (agranulocytosis). However, CLZ is more effective than other antipsychotics in the first or second treatment line [41].

The Cost Utility of the Latest Antipsychotic Drugs in Schizophrenia Study (CUtLASS 2) study showed that patients resistant to at least two antipsychotic drugs had significantly improved outcomes after one year on clozapine [42]. In TRS, memantine addition to clozapine significantly improved verbal and visual memory (*n* = 26) [43].

The phase 2E Clinical Antipsychotic Trials for Intervention Effectiveness (CATIE) study showed that clozapine had a better therapeutic response than risperidone and quetiapine. The researchers postulated that patients whose symptoms did not improve with a second-generation antipsychotic would benefit if prescribed clozapine rather than another second-generation antipsychotic drug [44].

#### Agranulocytosis and the Nitrenium Ion: Potential Leukocyte Autoimmune Biomarkers

**Figure 6:**
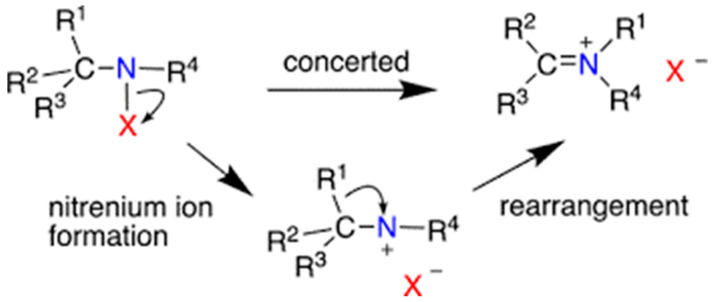
Nonaryl nitrenium ions and their rearrangements (computational studies after Falvey, 2018) [46].

Agranulocytosis is the most dangerous adverse effect linked to clozapine administration. Agranulocytosis/granulocytopenia induced by clozapine is not very common but can have a fatal outcome. The pathogenesis is not understood. Recent research postulates that this type of agranulocytosis is linked to an autoimmune response of the organism. The nitrenium ion can be activated biochemically by clozapine. The CYP3A4, CYP2D6, and myeloperoxidase system in white blood cells synthesize nitrenium ions (Figure 6). Therefore, the main component in the pathogenesis of clozapine-induced agranulocytosis might be a genetic aberration in the antigen genes of the leukocytes and some genes related to apoptosis [45].

A genetic study observed that reactive oxidative species could oxidize clozapine metabolites to nitrenium ions and concluded that this adverse reaction to clozapine is more likely a complex polygenic trait [47].

Agranulocytosis is diagnosed when the absolute neutrophil count is <100/mm^3^, associated with an infectious disease. A study on the Indian population found a 0.38% risk of agranulocytosis and neutropenia in patients treated with clozapine. The highest level was reached in the first six months after the initiation of treatment and remained significantly high for 18 months, with few cases reported after this period [48].

Another study, conducted in 2016, surprisingly concluded that neutropenia incidence during treatment with clozapine was not related to the drug itself and that patients diagnosed with schizophrenia and treated with other antipsychotics had the same risk of neutropenia. Blood counts were made every 25 days; after this period, the median was 124 days. After an average observation time of 9.2 years, of 201 patients on clozapine versus 410 patients with schizophrenia who had never been on clozapine, 34 cases of neutropenia were registered under clozapine; 24 patients had mild cases of neutropenia (1500–1900 neutrophils/mm^3^), which did not progress to agranulocytosis. The other 10 patients developed more severe neutropenia (500–1400 neutrophils/mm^3^); among them, only one progressed towards agranulocytosis. Three other patients discontinued clozapine, while six others remained on the drug for at least one year without any hematological side effects [49].

The risk of developing agranulocytosis is under 1% in CLZ patients, which may be independent of dosing [50,51], occurring in the first month of the treatment, up to six months; in this time span, it requires extensive blood absolute neutrophil counts [52]. The link between clozapine and agranulocytosis is still uncertain, with links between drug interactions, the immune system, and the genetic predisposition [53]. A study in 2015 analyzed the benefits of pharmacogenetic testing in patients at risk of clozapine-induced agranulocytosis. A lower genetic risk may benefit from a more “relaxed hematological monitoring” schedule [54]. Risk factors include old age, being female, genetics, and simultaneous treatment with drugs that generate agranulocytosis. Granulocyte colony-stimulating factor (G-CSF or GCSF) may stimulate the hematopoietic system to produce more white blood cells [55].

Clozapine-associated neutropenia is thought to occur due to selective neutrophil toxicity mediated by clozapine N-oxide metabolites [56] or an immune response mediated by a hapten-based mechanism [57] that occurred via an early exposure.

### 3.5. Clozapine Pseudoresistance

There are clinical situations that prove pseudo resistance to clozapine [58]. Pharmacodynamic factors cause treatment-resistant schizophrenia; the pseudo resistance is underlined by clinical or dependent on pharmacokinetics. The pseudo resistance might mask an inaccurate diagnosis, dose, or treatment duration; it might also be caused by insufficient medication levels in the serum, limited compliance, or comorbidities (including substance use) [59]. In many studies, the mortality rate of patients undergoing clozapine treatment proved lower than that of patients on first-generation antipsychotic drugs and atypical ones like quetiapine and risperidone [60].

The literature is scarce regarding clozapine use in later life; this particular approach is needed due to older people’s physical comorbidities and the increased risk of adverse effects; nevertheless, recent reviews highlight a definite benefit even in the elderly, with proper care and monitoring [61].

### 3.6. Clozapine Resistance and ECT Augmentation

In the population of psychiatric patients diagnosed with treatment-resistant schizophrenia, 40–70% of individuals are estimated to have incomplete or no response to clozapine, with low improvements on psychometric scores (lower than 20% from baseline) [62]. Among clozapine patients with schizophrenia, 12–20% will be ultra-resistant [20]. The term clozapine-resistant schizophrenia was introduced by Mouffak et al. [63]. The following criteria were proposed in defining this category of patients: Brief Psychiatric Rating Scale (BPRS) improvement less than 20% after a trial with clozapine of at least eight weeks, no stable period of proper social and occupational functioning for at least five years, Global Assessment of Functioning (GAF) score lower than 40, BPRS score higher than 45, a Clinical Global Impression (CGI) score higher than or equal to four, and a score of at least four on two out of four positive symptoms.

Twenty-eight studies were published on clozapine and ECT augmentation on the PubMed^®^ database, with 12 systematic reviews and meta-analyses, one randomized controlled trial, and three case series.

In addition to clozapine, ECT is an effective treatment in resistant schizophrenia spectrum disorders [64,65]; ECT’s practice still lacks consensual protocols. The first reported success in clozapine augmentation treatment by electroconvulsive therapy was first described in the early 1990s. Since then, this therapeutical combination has been used as a last treatment option, with excellent results showing clozapine’s superior efficacy compared to other antipsychotics in combination with ECT.

Clozapine and ECT are potent combinations in psychotic TRS; the other potent combination is flupenthixol augmented with ECT [66]. It showed a notable improvement in clinical and cognitive outcomes and decreased scores on the Positive and Negative Symptom Scale (PANSS) by 44% on ECT–CLZ in an acutely psychotic patient with TRS who responded to clozapine.

Side effects of bilateral ECT-augmented CLZ therapy may be delayed onset/protracted delirium and aspiration pneumonia, signaling the need for the careful monitoring of delirium in ECT augmentation on high-dose clozapine and/or the choice of unilateral ECT [67]. Nevertheless, CLZ posology should be reviewed when ECT treatment is initiated; as there is a lack of knowledge in this direction, more clinical trials on ECT–TRS are needed to confirm the effectiveness and safety [68].

### 3.7. Clozapine in Treatment-Refractory Bipolar Disorder

Treatment-refractory bipolar disorder (TRBD) can be defined as a bipolar disorder that does not respond to at least two courses of adequate different treatments [69].

In total, 543 studies were published on clozapine and bipolar disorder on the PubMed^®^ database, including 175 systematic reviews and meta-analyses, over the past ten years, with 24 bipolar disorder systematic reviews and meta-analyses and five randomized controlled trials.

It was concluded that clozapine could be useful for mood-related symptoms and rapid cycling patients and psychotic symptomatology associated with bipolar disorder. It could also improve the number of episodes necessitating hospital admission and the number of drugs in the therapeutical plan. Good results were also found in the area of social functioning, hetero-aggressivity, and suicidal thoughts.

Depression in schizophrenia has been a neglected field for some time; it was demonstrated that clozapine has an anti-suicidal effect not related to its antipsychotic action. Clozapine is exceptionally useful in reducing the risk of suicide in patients with schizophrenia [70]; the International Suicide Prevention Trial concluded that those properties could be as useful in bipolar disorder as they are in schizophrenia [71]. The rapid discontinuation of a CLZ regimen due to side effects in these patients may be followed by suicide [72].

Clozapine has been used to treat TRBD for over 30 years with excellent results. It positively influences suicidal ideation and aggressivity; only 1.5% of bipolar patients are prescribed CLZ [73]. In manic episodes, CLZ efficacy was similar to other antipsychotics or superior to other antipsychotics in treatment-resistant bipolar disorder (TRBD); however, it remains under-prescribed in these cases [74].

### 3.8. Side Effects during Clozapine Therapy

The frequent side effects associated with clozapine therapy (>10%) are cardiovascular: tachycardia orthostatic hypotension or hypertension, gastrointestinal—constipation, dyspepsia, nausea, sialorrhea, vomiting, or weight gain; nervous system-related: dizziness, drowsiness, insomnia, sedated state, vertigo, or fever.

Less common (below 10%) side effects are agranulocytosis, myocarditis, metabolic, seizures, sialorrhea, and pulmonary embolism. Other side effects may include fever, dizziness, headache, syncope, diaphoresis, nausea, vomiting, weight gain, sedation, sexual dysfunction, and urinary retention.

The black box warnings mention severe neutropenia, orthostatic hypotension, bradycardia, syncope, seizures, myocarditis, cardiomyopathy, mitral valve incompetence, and increased mortality in elderly patients with dementia-related psychosis [27].

#### 3.8.1. Metabolic Side Effects of Clozapine

Antipsychotic agents are linked to metabolic disorders, weight gain, diabetes mellitus, and dyslipidemia, leading to increased cardiovascular risk. Clozapine has the worst metabolic profile of all antipsychotics, mediated by an effect on the glucagon-like peptide (GLP-1) [14].

Olanzapine and clozapine determine the most metabolic disturbances; aripiprazole, brexpiprazole, and cariprazine had the fewest metabolic side effects, making them the safest to use. The risk factors for adverse metabolic reactions included: high body mass index (BMI) at the beginning of the treatment, male gender, and non-white ethnicity; the improvement of psychiatric symptoms is associated with dire metabolic side effects; in the context of the increased use of antipsychotic drugs at a global level, the specific BMI and adverse metabolic reactions must be monitored and promptly treated during the clinical course of the psychiatric disease [75].

The dysmetabolic side effects of clozapine differ based on gender differences. Their prevalence is more frequent among females, who reach a higher plasmatic clozapine concentration—on average, 17% greater—than the plasmatic concentration in men, but BMI and the plasmatic glucose levels of females were higher than in men [76]. Since clozapine is a lipophilic drug, and females usually have more adipose tissue than men, it was expected that women would show lower concentrations in their plasma at a given dose.

More studies show the opposite; the reason underlying this might be the gender differences in pharmacokinetics, such as faster renal clearance in men [77]. No significant gender differences were found regarding the concentration of norclozapine (the major active metabolite of clozapine); this further adds to the observation that women are at higher risk of clozapine accumulation and side effects (because the ratio between CLZ and norclozapine is increased).

Clozapine, like other antipsychotic drugs (quetiapine, haloperidol, trifluoperazine, risperidone, aripiprazole, and olanzapine) [78], significantly increases body weight by ≥7% from the baseline. Obesity is associated with a series of comorbidities that decrease life expectancy. The pharmacogenetic markers in CYP2C19, leptin, leptin receptor and HTR2C receptors predict higher metabolic complications and BMI [79].

However, antidepressant users may have higher food intake compared to non-users [80]. Certain studies showed that among non-diabetic patients with metabolic anomalies, the use of clozapine and metformin could reduce their body weight and reverse metabolic abnormalities [81]. The Controlled Trial of co-commencement of METformin (COMET) protocol for a randomized controlled trial with metformin therapy as an adjunctive treatment to reduce weight gain and metabolic syndrome in patients with schizophrenia newly on clozapine was recently initiated [82].

#### 3.8.2. Clozapine Risk of Pulmonary Embolism and Pneumonia

Venous thromboembolism is rare among the side effects of clozapine, but compared to other atypical antipsychotic drugs, the risk is more elevated [83]. The onset of pulmonary embolism in clozapine users is linked to other cardiometabolic risk factors like age, obesity, and smoking in schizophrenic patients.

There are many theories regarding the etiology of pulmonary embolism in patients treated with clozapine. Another hematological side effect might be at the root of the venous thromboembolism in the patients treated with clozapine—the increase in adhesion and aggregation of blood platelets [84].

A recent in vitro study revealed that clozapine enhances the risk of developing thrombosis in patients on CLZ, by reducing the coagulation time and fibrin fibers’ thickness, developing a thrombogenic pattern in a dose-dependent manner [85].

Patients diagnosed with schizophrenia are at higher risk of obesity and cardiovascular diseases; this observation is another pathophysiological explanation of the increased risk of pulmonary embolism [86] and aspiration pneumonia [87]. Sedation is another significant and widespread side effect of clozapine administration related to body mass index (BMI) modification and can be linked with sedentary and venous stasis development.

Case studies of pulmonary embolism associated with clozapine treatment concluded that this side effect, though rare, is lethal. In general, this adverse reaction has an early onset and is not dependent on the dose, bringing into discussion the opportunity of prophylactic measures for venous thromboembolism six months after initiating clozapine [88,89].

#### 3.8.3. Clozapine and Seizure Risk

Clozapine is the most frequent atypical antipsychotic agent associated with seizures. This lowers the seizure threshold depending on the dose: 300–600 mg/day resulted in a seizure prevalence of 1.8%, which increased to 4.4% at doses higher than 600 mg/day [90].

One proposed etiological mechanism of clozapine-induced seizures claims that this drug has an affinity for the mesolimbic dopamine receptors, while typical antipsychotics usually bind to the striatonigral dopamine receptors. The mesolimbic structures represent a frequent seizure onset site; this might be an argument for the high epileptogenicity of clozapine compared to other antipsychotic medications [91].

Given the high efficiency of clozapine in managing psychiatric disorders, convulsive episodes are not an indication for stopping treatment in most cases. The available antiepileptic drugs such as valproate, topiramate, or lamotrigine proved effective in treating this particular side effect [92].

A retrospective study examined seven years of the medical history of 222 patients after starting clozapine treatment to evaluate seizure incidence before and after the treatment. The results showed that 6% of patients had seizures, a side effect correlated with the clozapine dose [93].

Some clinicians recommend anticonvulsant therapy in association with clozapine. However, it was not proved that this brought about a positive outcome in every circumstance. Some authors postulate that the plasma concentration of clozapine (1300 ng/mL) is a better predictor than the dosage when it comes to seizure risk. In addition, it has to be considered that the concentration of clozapine is correlated with age, gender, BMI, and genotype variations; anticonvulsants associated with clozapine may lead to a higher risk of severe side effects or may interfere with the therapeutic response, so clinical psychiatrists must take into consideration potential drug interactions [94].

### 3.9. Clozapine and COVID-19 Infection

A “clozapine” and “COVID 19” search retrieved 22 results in a PubMed^®^ database search and 26 results in Google Scholar^®^, from which two reviews considered topics of toxicity and side effects, management of clozapine monitoring and therapy during the SARS-COV-2 quarantine, COVID-19 patients, and clozapine and agranulocytosis—including the International Prospective Register of Systematic Reviews (PROSPERO)—CRD42020178819 trial investigating the influence of COVID-19 on mental health patients [95].

Patients with COVID-19 infection frequently experience lymphopenia but seldomly neutropenia [90]. COVID-19 infection may cause clozapine intoxication by dramatically increasing serum clozapine levels, affecting the cytokine release, and downregulating the metabolism of clozapine in the CYP450 system/CYP 1A2 [96], as revealed by recent studies and case reports [97,98]. SARS-CoV2 in CLZ therapy has increased pneumonia risk and clozapine toxicity risk, even the need for intervention in a critical care unit, and the disruption of CLZ therapy by COVID-19 induced lymphopenia [99,100,101,102].

Nevertheless, the therapy must be continued with the following recommendations [102,103]:

(1) The neutrophil count may be reduced to every three months, with a dispensation of up to a 90 day supply on receipt) for people fulfilling the following criteria: • continuous clozapine treatment for more than one year or • have never had a neutrophils count below 2000/µL or • safe and practical access to ANC testing.
(2) Patients on clozapine and with no COVID-19 symptoms (cough, fever, chills, sore throat, myalgia, fatigue, other flu-like symptoms) need an immediate in-person or distance medical evaluation, involving a complete blood count including neutrophils, according to the local protocols [104].
(3) If patients on clozapine become COVID-19 symptomatic, they may be required to decrease by half the dose of clozapine up to three days after the fever has passed, a point at which clozapine can be gradually increased to the pre-fever dose. Where available, clozapine levels could back up the clinical decision.

In a recent cohort study on 6309 participants, of whom 102 were positive for SARS-CoV-12, clozapine treatment was linked with an increased risk of COVID-19 infection, more than other antipsychotics, an association that needs further research to be confirmed [105].

Psychiatrists and healthcare providers involved in monitoring the absolute neutrophil count (ANC) and dispensing the prescription must be aware of the increased risks in clozapine-treated patients with COVID-19. They must communicate and strengthen the monitoring of side effects, including their duration, as the full impact of the COVID-19 pandemic is still unknown [106].

### 3.10. Safety of Clozapine in Pregnancy

**Figure 7:**
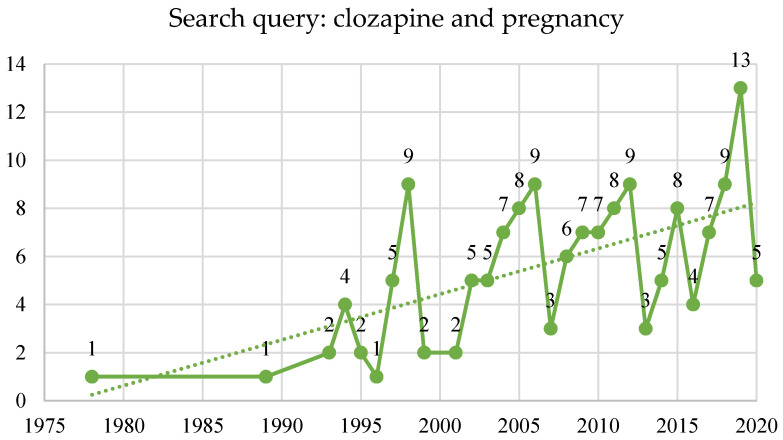
Search query: clozapine and pregnancy.

On the clozapine in pregnancy topic, 129 studies were published in the PubMed^®^ database beginning in 1978, with 12 systematic reviews and meta-analyses, and two randomized controlled trials (Figure 7) [107].

Mehta et al. showed that there were not many data about clozapine treatment during pregnancy. The lack of control regarding the treatment doses, duration, and exposure of the fetus does not allow us to get an accurate picture of the clozapine effects in utero [107].

The penetration ratios (antipsychotic concentrations in the target matrix (i.e., amniotic fluid, umbilical cord blood, or breast milk)/maternal concentration) in the amniotic fluid were estimated with a mean of 0.56 in the range of 0.31–0.82 for clozapine, while in the breast milk there was a mean of 3.19, within the range of 2.79–4.32, indicating the need for measuring antipsychotic concentrations in maternal blood to estimate fetal/infant exposure [108].

Several case reports showed CLZ teratogenic effects and associated disorders, ranging from congenital malformations to metabolic and neurological disorders and unwanted side effects for pregnant women [109]. In addition, case reports show that clozapine increases the risk of developing gestational diabetes [110,111]. However, this was put into doubt by a 2020 paper that found no significant relationship between antipsychotic drugs, including clozapine, and the risk of gestational diabetes mellitus (GDM) but indicated a higher risk of fetal macrocephaly [112].

A follow-up study found malformations in the children of 4.2% of mothers treated with clozapine during pregnancy [113]. Other papers reported that seven out of 84 women under clozapine treatment suffered spontaneous abortions. There were also mentions of shoulder dystocia [114,115], atrial septum defect, ectopic anus, floppy infant syndrome, electro encephalogram (EEG) abnormalities [116], seizures, and digestive tract-related disorders.

Regarding the long-term effect on children born to mothers treated with clozapine during pregnancy, we found a single study that concluded that children had a lower score in the Bayley III scale of adaptative behavior than those whose mothers received treatment with other atypical antipsychotics [117].

### 3.11. Safety of Clozapine in Very Early-Onset Schizophrenia or Childhood-Onset Schizophrenia

Early-onset schizophrenia occurs before age 18; very early-onset schizophrenia (EOS) or childhood-onset schizophrenia (COS) begins at under 13 years old and is extremely rare [118]. It is well known that EOS has a poorer prognosis and more severe symptoms, as it affects the individual before the brain and personality are fully developed. The risks and benefits of clozapine administration in adults have been well assessed, as reviewed above, but clinicians are reluctant to prescribe clozapine for younger psychiatric patients.

**Figure 8:**
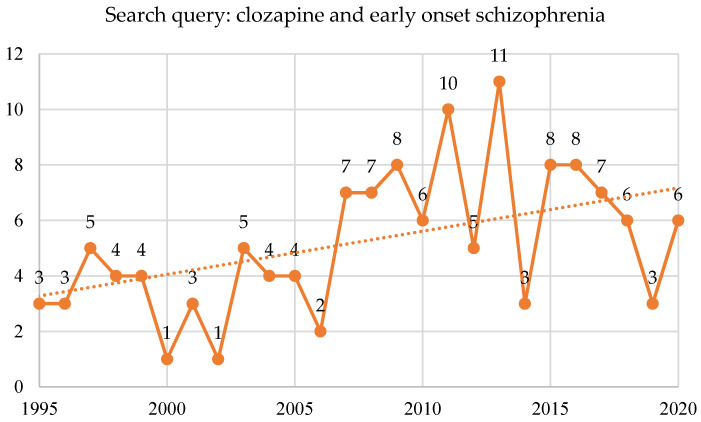
Search query: clozapine and early-onset schizophrenia in PubMed^®^—total studies.

A total of 125 studies were published on clozapine and early-onset schizophrenia or childhood-onset schizophrenia in the PubMed^®^ database beginning in 1994, with six randomized controlled trials, six systematic reviews, and three meta-analyses (Figure 8).

The present protocols advise against prescribing clozapine in early-onset schizophrenia after two trials of different antipsychotics showed no improvement and to schedule regular follow-ups to check for adverse reactions. As in adults, clozapine also showed its superior efficacy in treatment-resistant early-onset schizophrenia. Clinical trials improved up to 69% in assessments with the Brief Psychiatric Rating Scale maintained up to nine years [119]. More than 90% of the patients complained of sedation and sialorrhea. Enuresis, intestinal transit disorders, weight gain, and EEG abnormalities were reported by 10–60% of patients; 1–30% complained of akathisia, blood pressure abnormalities, and tachycardia, and 6–15% developed transient neutropenia. Agranulocytosis incidence was under 0.1%. In 8–22% of the cases, there were metabolic disorders, but diabetes had a less than 6% incidence; 3–6% of the patients discontinued the use of clozapine.

The scientific data prove that clozapine is an effective and safe treatment for refractory early-onset schizophrenia [120].

### 3.12. Clozapine Use in Romania

**Figure 9:**
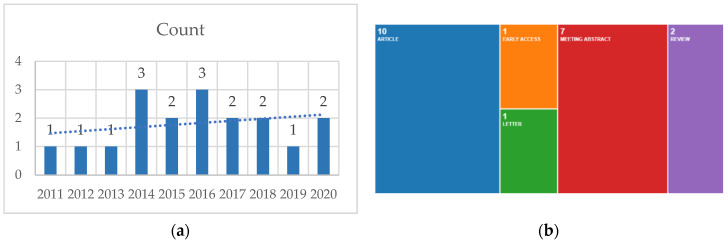
The data search on clozapine and Romania search query (**a**) on PubMed^®^—all-time papers published by year (*n* = 18) (**b**) Web of Science^®^—fourteen papers during the last ten years, four reviews and ten articles (one early access).

The data search on clozapine and Romania retrieved 14 results on PubMed^®^ and five on Web of Science^®^; no clinical trials or RCTs were found on PubMed^®^, with one on Web of Science^®^ (Figure 9).

One clinical trial [121] has evaluated the safety and effectiveness of CLZ in patients with a lack of response to other antipsychotics, showing rapid clozapine titration to be safe and effective in the therapy of schizophrenia, with an initiation time of 7.1 ± 4.8 days [122]; however, a study performed in Romania showed that the rapid titration of clozapine increases the risk of myocarditis in the first 2–8 weeks after the initiation of the therapy [123].

A cross-sectional study in Brasov, Romania [123], was conducted for four years on 115 patients with schizophrenia (39.7 ± 11.1 years; males: 59%) involuntarily admitted and restrained due to violence; the study suggests an early CLZ anti-aggressive effect in highly problematic patients, an outcome previously showed in an efficacy study on 337 patients [124].

A study conducted by Ifteni et al. (2017) on the clinical efficacy of clozapine in bipolar disorder showed that switching from clozapine to another antipsychotic may increase the risk of relapse [125].

Teodorescu et al. (2020) published a recent paper on clozapine and treatment-refractory aggressive behavior based on a study of 504 patients admitted to the Clinical Hospital of Psychiatry and Neurology of Brasov; CLZ was effective and safe in cases of patients with treatment-refractory aggressive behavior [126,127].

More studies are needed to highlight the safety profiles and use in specific population groups due to the Romanian geographical population’s specific profile.

### 3.13. Publication Bias

There were insufficient studies to test for publication bias.

## 4. Discussion

There is an urgent need to enhance access to clozapine for people with TRS at the worldwide level [128]; however, four in 10 people with TRS fail to respond to clozapine, suggesting that 12–20% of all people with schizophrenia will be ultra-resistant [129].

- The genetic vulnerability is correlated with metabolic side effects and postulates predictors of side effects’ severity; individuals who metabolize clozapine more slowly are at higher risk of this type of disorder. DiGeorge syndrome (22q11.2 deletion syndrome) is seen in up to 41% of schizophrenia spectrum disorders, mostly treatment-resistant schizophrenia. The medical comorbidities may complicate pharmacotherapy administration; psychiatrists should adjust/personalize clozapine doses for each patient based on pharmacogenetic testing as a potential biomarker in the severity of potential side effects.
- Agranulocytosis and neutropenia occur in less than 1% of patients who take clozapine; they are at the highest level in the first six months after the initiation of treatment, and remain significantly high for 18 months; patients diagnosed with schizophrenia treated with other antipsychotics had the same risk of neutropenia, while patients with a lower genetic risk may need less frequent hematological monitoring. Granulocyte colony-stimulating factor therapy (i.e., filgrastim) can reduce the impact of agranulocytosis.
- Olanzapine and clozapine lead to the most metabolic disturbances, which differ based on gender, and are more frequent among females.
- Pulmonary embolism associated with clozapine treatment, though rare, is lethal (a mortality rate of 36.36%), affirming the need for prophylactic measures for venous thromboembolism for six months after initiating clozapine.
- Convulsive episodes are not an indication for stopping treatment; 6% of patients had seizures, with the incidence increasing with the dose, so the plasma concentration of clozapine (1300 ng/mL) is a better predictor than dosage, and anticonvulsants associated with clozapine may lead to a higher risk of severe side effects or may interfere.
- There are not many data about clozapine treatment during pregnancy, the risk of developing gestational diabetes, spontaneous abortions, teratogenic effects, associated disorders (4.2% malformations), higher risk for macrocephaly, and a lower score in Bayley III scale of adaptative behavior.
- Clozapine remains an effective and safe treatment for refractory early-onset schizophrenia, with an improvement of up to 69% in assessments with the Brief Psychiatric Rating Scale; the agranulocytosis incidence was under 0.1%.
- Few papers are published in indexed databases about Romania’s topic; more studies are needed to highlight the experience and pharmacogenetic characteristics of this specific geographical population in relation to clozapine therapy.
- COVID-19 infection may enhance clozapine toxicity by significantly increasing serum clozapine levels by the CYP 450 system, generating an increased risk of pneumonia or even the need for intervention in a critical care unit. Nevertheless, the therapy must be continued with the proper monitoring of the white blood count; patients with COVID-19 may require a decrease in the clozapine dose by half until three days after the fever subsides before gradually re-establishing the initial dosage. Psychiatrists and healthcare providers must be aware of the increased risks in clozapine-treated patients with COVID-19 and must communicate and strengthen the monitoring of side effects.

## 5. Limitations

Within the study, we selected CT/RCT from the PubMed and Web of Science Core Collection databases, searched by title and abstract topic; our study did not analyze papers present in other databases.

## 6. Conclusions

Clozapine has been known and used for an extended period, but in the past three or four decades, there has been a failure to generate effective novel psychopharmaceuticals. Due to the limited prospects for new, more effective antipsychotics in the short to medium term [124], there is a need to maximize access to clozapine therapy and investigate therapies that mitigate the side effects of CLZ in resistant cases.

The genetic vulnerability postulates predictors of adverse reactions’ severity, so clozapine doses should be personalized for each patient based on pharmacogenetic testing; patients with a lower genetic risk may have less frequent hematological monitoring.

The pulmonary embolism associated with clozapine has a mortality rate of 36.36%, so prophylactic measures for venous thromboembolism for six months after initiating therapy are mandatory. The convulsive episodes are not an indication for stopping treatment; side effect (s.e.) incidence increases with dose, so the plasma concentration of clozapine (1300 ng/mL) is a better s.e. predictor than the dosage.

Clozapine improves treatment-refractory early-onset schizophrenia by up to 69%, as assessed by the Brief Psychiatric Rating Scale (BPRS); more pharmacogenetic studies of Romanian schizophrenic patients are needed in relation with clozapine therapy in order to define more precise safety margins.

COVID-19 infection may enhance clozapine toxicity, generating an increased risk of pneumonia, so therapy must be continued with the proper monitoring of the white blood count and a decrease in the clozapine dose by half until three days after the end of the fever. Psychiatrists and healthcare providers must act together to choose the proper treatment and doses to achieve results in clozapine-treated patients with COVID-19.

Since in the past four decades research has failed to generate effective novel psychopharmaceuticals [130], there is an urgent need to enhance access to clozapine for people with TRS worldwide. Nowadays, progress in pharmacogenetic research, discoveries in the area of endocrinology, genetic testing, and other interdisciplinary approaches offer psychiatrists the chance to use this drug at its highest potential, in a personalized manner for every patient, minimizing the adverse side effects and perhaps decreasing the rate of clozapine resistance by correctly identifying the clinical situation and the neurobiology of the resistance.

## Supplementary Materials

The following are available online at https://www.mdpi.com/2076-3425/10/11/840/s1, Table S1: Database search results by numbers, Table S2: Pharmacogenetic biomarkers in clozapine treatment—selected papers.

## Abbreviations

ADHD	Attention-Deficit/Hyperactivity Disorder
ANC	Absolute Neutrophil Count
BAF	Bipolar Affective Disorder
BMI	Body Mass Index
BPRS	Brief Psychiatric Rating Scale
RCT	Randomized Controlled Trial
CCS	Clinical Case Series
C/D ratio	Concentration-to-Dose Ratio
COS	Childhood-Onset Schizophrenia
ECT	Electroconvulsive Therapy Augmentation
ECG	Electro Cardiogram
EEG	Electro Encephalogram
EOS	Early-Onset Schizophrenia
GDM	Gestational Diabetes Mellitus
PANSS	Positive and Negative Symptom Scale
PRISMA	Preferred Reporting Items for Systematic Review and Meta-Analysis
PTE	Pulmonary Thrombo Embolism
QT	Time from the start of the Q wave to the end of the T wave on ECG
RCT	Randomized Controlled Trial
SARS-CoV-2	Severe Acute Respiratory Syndrome Coronavirus 2
TRS	Therapy-Resistant Schizophrenia
TRBD	Treatment-Resistant Bipolar Disorder
WHO	World Health Organization
