# Supplementary material for: Clozapine: An Updated Overview of Pharmacogenetic Biomarkers, Risks, and Safety—Particularities in the Context of COVID-19
Source: Brain Sci. 2020 Nov 11;10(11):840. doi: 10.3390/brainsci10110840 (PMC7697202; doi:10.3390/brainsci10110840)
Supplement: Supplementary file 1 [file brainsci-10-00840-s001.zip › Figure 1 CLZ.pdf]

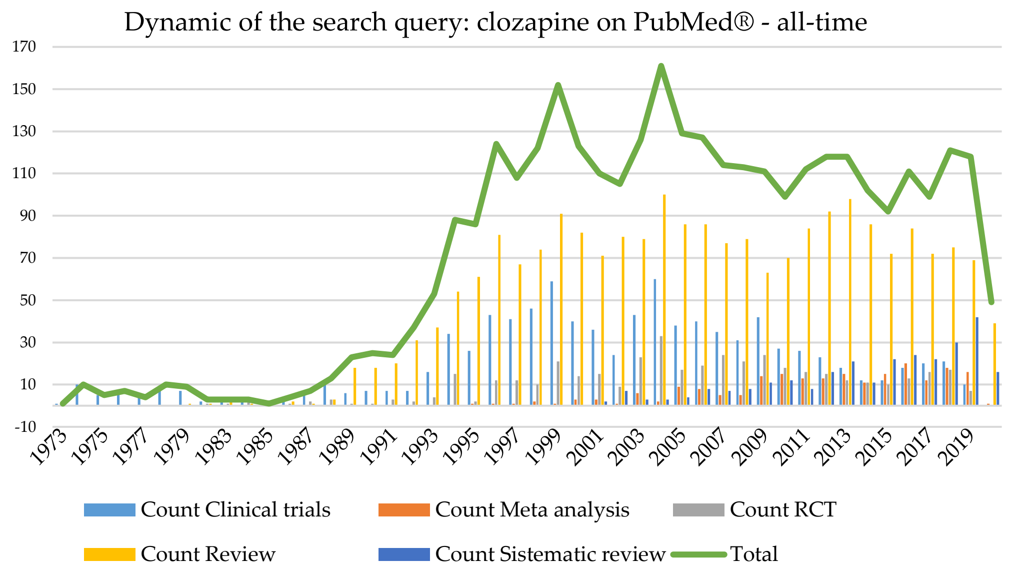

(a)

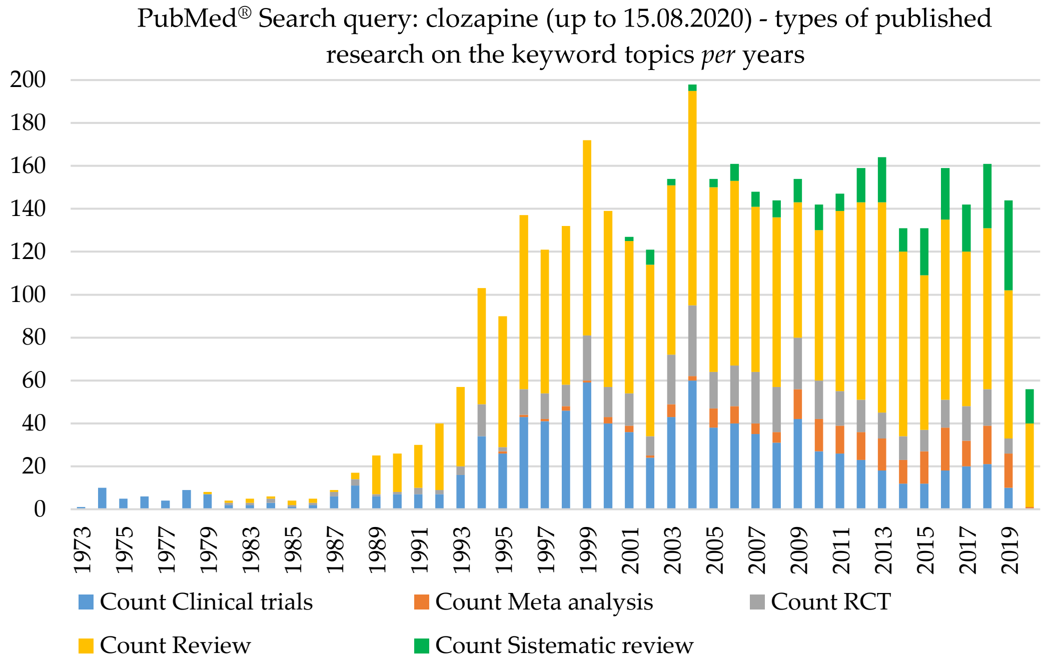

(b)

Figure no. 1 Systematic search of keyword “clozapine” on PubMed® database (all-time topic) retrieved over 4000 results, showing a persistent interest in the drug (a) total (b) by type of study
