# Supplementary material for: Clozapine: An Updated Overview of Pharmacogenetic Biomarkers, Risks, and Safety—Particularities in the Context of COVID-19
Source: Brain Sci. 2020 Nov 11;10(11):840. doi: 10.3390/brainsci10110840 (PMC7697202; doi:10.3390/brainsci10110840)
Supplement: Supplementary file 1 [file brainsci-10-00840-s001.zip › Table 1 CLZ database search results.pdf]

Table 1. Databases search results by numbers

| Main keyword | Secondary Keyword | PubMed® (n*) | | Web of Science®(n*) | |
|---|---|---|---|---|---|
| | | 10 years | Refined | 10 years | Refined |
| clozapine | Treatment resistant schizofrenia | 60 | 19 | 148 | 69 |
| clozapine | Resistance | 4 | 3 | 21 | 5 |
| clozapine | Bipolar disorders | 4 | 3 | 38 | 19 |
| clozapine | Treatment resistant bipolar disorder | 2 | 2 | 7 | 5 |
| clozapine | Side effects | 6 | 6 | 21 | 8 |
| clozapine | Agranulocytosis | 12 | 12 | 68 | 33/ |
| clozapine | Obesity or metabolic | 3+14 | 3+14 | 12/193 | 10/53 |
| clozapine | Pharmacogenetic | 5 | 4 | 6 | 6 |
| clozapine | Pulmonary embolism | 3 | 3 | 7 | 4 |
| clozapine | Seizure | 8 | 6 | 23 | 11 |
| clozapine | COVID 19 | 10 | 9 | 5 | 3 |
| clozapine | Pregnancy | 2 | 2 | 7 | 4 |
| clozapine | Early-onset/childhood-onset schizophrenia | 3 | 3 | 12 | 6 |
| clozapine | Romania | 5 | 3 | 3 | 3 |
| | **Total** | **141** | **92** | **571** | **239** |

*n-number of search results
