# Supplementary material for: Clozapine: An Updated Overview of Pharmacogenetic Biomarkers, Risks, and Safety—Particularities in the Context of COVID-19
Source: Brain Sci. 2020 Nov 11;10(11):840. doi: 10.3390/brainsci10110840 (PMC7697202; doi:10.3390/brainsci10110840)
Supplement: Supplementary file 1 [file brainsci-10-00840-s001.zip › Table 2 CLZ Pharmacogenetic markers.pdf]

Table 1 Pharmacogenetic biomarkers in clozapine treatment

Reference	Article Title	Source Title	Abstract
Girardin, Francois R.; & al. 2019	Cost-effectiveness of HLA-DQB1/HLA-B pharmacogenetic-guided treatment and blood monitoring in US patients taking clozapine	PHARMACOGENOMICS JOURNAL	Less than 1% of adult patients with schizophrenia taking clozapine develop agranulocytosis, and most of these cases occur within the first weeks of treatment. The human leukocyte antigen (HLA) region has been associated with genetic susceptibility to clozapine-induced agranulocytosis (single amino acid changes in HLA-DQB1 (126Q) and HLA-B (158T)). The current study compared current practices using two genotype-guided strategies: (1) HLA genotyping followed by clozapine, with ANCM only for patients who tested positive for one or both alleles (genotype-guided blood sampling); (2) HLA genotyping followed by clozapine for low-risk patients and alternative antipsychotics for patients who tested positive (clozapine substitution scheme). Up to a decision threshold of $3.9 million per quality-adjusted life-year (90-fold the US gross domestic product per capita), the base-case results indicate that compared with current ANCM, genotype-guided blood sampling prior to clozapine initiation appeared cost-effective for targeted blood monitoring only in patients with HLA susceptibility alleles. Sensitivity analysis demonstrated that at a cost of genotype testing of up to USD700, HLA genotype guided blood monitoring remained a cost-effective strategy compared with either current ANCM or clozapine substitution.
Numata, Shusuke & al. 2018	Clozapine Pharmacogenetic Studies in Schizophrenia: Efficacy and Agranulocytosis	FRONTIERS IN PHARMACOLOGY	Clozapine is an efficacious atypical antipsychotic for treatment-refractory schizophrenia. Clinical response and appearance of adverse events vary among individual patients receiving clozapine, with genetic and non-genetic factors potentially contributing to individual variabilities. The studies on clozapine pharmacogenetics have been extensively examined the clinical response and agranulocytosis. While there is still limited evidence on clozapine efficacy, recent genome-wide studies provide further evidence of the involvement of the human leukocyte antigen (HLA) region in clozapine-induced agranulocytosis.
Verbelen, Moira & al. 2017	Establishing The Characteristics Of An Effective Pharmacogenetic Test For Clozapine Induced Agranulocytosis	EUROPEAN NEURO PSYCHOPHARMACOLOGY	Clozapine is the only evidence-based therapy for treatment-resistant schizophrenia, but it induces agranulocytosis, a rare but potentially fatal hematological adverse reaction, in less than 1% of users. A pharmacogenetic test is clinically useful if it identifies a group of patients for whom the agranulocytosis risk is low enough to alleviate monitoring requirements. Assuming a genotypic marker stratifies patients into a high-risk and a low-risk group, we explore the relationship between test sensitivity, group size and agranulocytosis risk. High sensitivity minimizes the agranulocytosis risk in the low-risk group and is essential for clinical utility, in particular in combination with a small high-risk group.
Verbelen, M. & al. 2015	Establishing the characteristics of an effective pharmacogenetic test for clozapine-induced agranulocytosis	PHARMACO -GENOMICS JOURNAL	Clozapine is the only evidence-based therapy for treatment-resistant schizophrenia, but it induces agranulocytosis, a rare but potentially fatal hematological adverse reaction, in less than 1% of users. To improve safety, the drug is subject to mandatory hematological monitoring throughout the course of treatment, which is burdensome for the patient and one of the main reasons clozapine is underused. Therefore, a pharmacogenetic test is clinically useful if it identifies a group of patients for whom the agranulocytosis risk is low enough to alleviate monitoring requirements. Assuming a genotypic marker stratifies patients into a high-risk and a low-risk group, we explore the relationship between test sensitivity, group size and agranulocytosis risk. Study proposed test of HLA-DQB1 6672G>C has high specificity, but low sensitivity fails to reduce the agranulocytosis risk in the LR group sufficiently that monitoring could be reduced or ceased.
Spencer, Benjamin W. J & al., 2013	Opening Pandora's box in the UK: a hypothetical pharmacogenetic test for clozapine	PHARMACOGENOMICS	Clozapine is a uniquely efficacious antipsychotic drug in treatment-resistant schizophrenia. Its use is restricted due to adverse effects including a rare but dangerous reduction in neutrophils (agranulocytosis) and the mandatory hematological monitoring this entails in many countries. This paper is a review on the statistical, ethical and legal issues arising from a hypothetical pharmacogenetic test for clozapine, using the UK as an exemplary case for consideration. Key findings include: a consideration of the probabilistic results that a pharmacogenetic test may return; the impact on drug licensing; and the potential for pharmacogenetic tests for clozapine being used without consent under the UK's legal framework. If any such test is to have a real impact in patient care, licensing changes must follow. Consideration as to the legal and ethical issues surrounding pharmacogenetic testing for treatment with clozapine needs to be made in advance.
Rajkumar, A. P & al., 2012	Outcome definitions and clinical predictors influence pharmacogenetic associations between HTR3A gene polymorphisms and response to clozapine in patients with schizophrenia	PSYCHOPHARMACOLOGY	Pharmacogenetics of schizophrenia has not yet delivered anticipated clinical dividends. Clinical heterogeneity of schizophrenia contributes to the poor replication of the findings of pharmacogenetic association studies. Functionally important HTR3A gene single-nucleotide polymorphisms (SNPs) were reported to be associated with response to clozapine. The aim of this study was to investigate how the association between HTR3A gene SNP and response to clozapine is influenced by various clinical predictors and by differing outcome definitions in patients with treatment-resistant schizophrenia (TRS). We recruited 101 consecutive patients with TRS, on stable doses of clozapine, and evaluated their HTR3A gene SNP (rs1062613 and rs2276302), psychopathology, and serum clozapine levels and employed appropriate multivariate statistics to calculate allelic and genotypic association, accounting for the effects of various clinical variables. T allele of rs1062613 and G allele of rs2276302 were significantly associated with good clinical response to clozapine ($p = 0.02$). However, varying outcome definitions make these associations inconsistent. rs1062613 and rs2276302 could explain only 13.8 % variability in the responses to clozapine, while combined clinical predictors and HTR3A pharmacogenetic association model could explain 38 % variability. The results of pharmacogenetic studies in schizophrenia depend heavily on their outcome definitions and that combined clinical and pharmacogenetic models have better predictive values. Future pharmacogenetic studies should employ multiple outcome definitions and should evaluate associated clinical variables.
