# Supplementary figures and images for: Clozapine: An Updated Overview of Pharmacogenetic Biomarkers, Risks, and Safety—Particularities in the Context of COVID-19
Source: Brain Sci. 2020 Nov 11;10(11):840. doi: 10.3390/brainsci10110840 (PMC7697202; doi:10.3390/brainsci10110840)

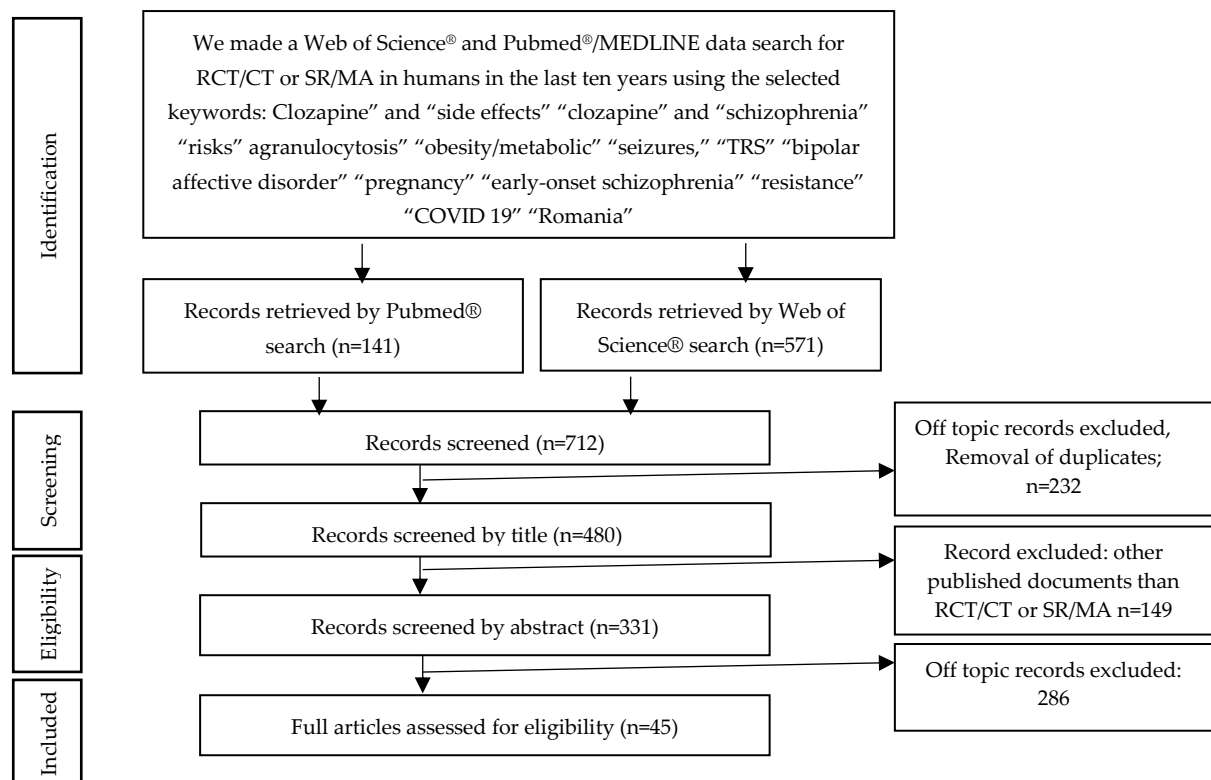

Figure no. 2 PRISMA diagram describing systematic search and study selection process.

Supplement: Supplementary file 1 [file brainsci-10-00840-s001.zip › Figure no. 2 PRISMA diagram .pdf]
